# Efficacy of three novel drugs in the treatment of heart failure: A network meta-analysis

**DOI:** 10.1097/MD.0000000000029415

**Published:** 2022-07-22

**Authors:** Lin Luo, Xu Yang, Kai Tang, Jianli Wu, Dejin Li, Jiuju Ran, Li Zhang, Dan Wang, Dan Zhao, Min Yu, Anfang Chen, Maya Saranathan

**Affiliations:** a Department of Cardiovascular, The First People's Hospital of Shuangliu District, Chengdu, China; b Department of Ophthalmolgy, Sichuan Academy of Medical Sciences, Sichuan Provincial People's Hospital, Chengdu, China.

**Keywords:** angiotensin receptor neprilysin inhibitors, efficacy, heart failure, network meta-analysis, novel heart failure drugs, sodium-glucose cotransporter 2 inhibitors, soluble guanylate cyclase stimulators

## Abstract

Angiotensin receptor neprilysin inhibitors (ARNI), sodium-glucose cotransporter 2 inhibitors (SGLT2i), soluble guanylate cyclase stimulators (sGCs), and the traditional golden triangle standard-of-care (SOC) are effective drugs for heart failure. We aimed to assess the efficacy of 4 interventions in these patients.

**Methods:**

PubMed, The Cochrane Library, Embase, and Web of Science databases were electronically searched to collect randomized controlled trials of 3 novel drugs in the treatment of heart failure from inception to September 1st, 2021. Two reviewers independently screened literature, extracted data, and assessed the risk bias of included studies. Stata 16.0 software was used for network meta-analysis.

**Results:**

A total of 17 randomized controlled trial involving 38,088 patients were included. The results of network meta-analysis: in terms of heart failure rehospitalization rate, 3 novel drugs lower than SOC [ARNI (OR = 0.77, 95% CI: 0.71–0.83), SGLT2i (OR = 0.70, 95% CI: 0.63–0.77), sGCs (OR = 0.88, 95% CI: 0.78–0.99)], and SGLT2i was also lower than sGCs (OR = 0.79, 95% CI: 0.68–0.93). In terms of all-cause mortality, ARNI was lower than SOC (OR = 0.81, 95% CI: 0.66–0.99). In terms of cardiovascular mortality, ARNI and SGLT2i was lower than SOC (ARNI [OR = 0.80, 95% CI: 0.70–0.92], SGLT2i [OR = 0.87, 95% CI: 0.76–0.99]). In terms of rates of cardiovascular death or heart failure rehospitalization, 3 novel drugs lower than SOC (ARNI [OR = 0.76, 95% CI: 0.71–0.82], SGLT2i [OR = 0.76, 95% CI: 0.70–0.82], sGCs [OR = 0.87, 95% CI: 0.78–0.97]). In terms of Kansas city cardiomyopathy questionnaire score, ARNI and SGLT2i was superior to SOC (ARNI [MD = 1.43, 95% CI: 0.43–2.42], SGLT2i [MD = 1.88, 95% CI: 1.12–2.65]). In terms of N-terminal pro-B-type natriuretic peptide outcome indexes, SGLT2i was superior to SOC (MD = −134.63, 95% CI: −237.70 to −31.56). The results of Surface under the cumulative ranking sequencing: in terms of heart failure rehospitalization rate and rates of cardiovascular death or heart failure rehospitalization, the ranking was SGLT2i>ARNI>sGCs>SOC. in terms of all-cause mortality and cardiovascular mortality, the ranking was ARN>SGLT2i>sGCs>SOC. in terms of Kansas city cardiomyopathy questionnaire score and N-terminal pro-B-type natriuretic peptide outcome indexes, the ranking was SGLT2i>ARN>SOC.

**Conclusions:**

The available evidence suggests that all 3 novel heart failure drugs can improve the prognosis of heart failure. ARNI may be the most effective in reducing mortality, SGLT2i may be the most effective in improving quality of life, while sGCs may be inferior to ARNI and SGLT2i.

## 1. Introduction

Heart failure is the main manifestation of the advanced stage of cardiovascular disease, and its mortality and rate of rehospitalization remain high for a long time. According to data, the prevalence rate of heart failure in Europe and America is 1.5% to 2.0%,^[1]^ whereas it is around 0.9% among Chinese adults.^[2]^ In recent years, as China has entered an aging country, the prevalence of heart failure has increased, and heart failure mortality in all ages has increased more than other cardiovascular illnesses in the same period.^[3]^ Optimizing the treatment of heart failure is extremely important. Traditional golden triangle therapy standard-of-care (SOC) includes angiotensin-converting enzyme inhibitors (ACEI)/angiotensin receptor blockers (ARB), β-receptor antagonists, and mineralcorticoid recept antagonists (MRA). This treatment option is still widely recognized. Both the Chinese guideline of 2018 and the European and American guidelines of 2021 have recommended as the basic therapy for heart failure (I, A).^[4–6]^ Various national guidelines offer angiotensin receptor neprilysin inhibitor (ARNI) as an alternative to ACEI for individuals who still have symptoms after undergoing the SOC regimen (I, B).^[4–6]^ With or without diabetes mellitus, the 2021 European Society of Cardiology guideline recommends the use of sodium-glucose cotransporter 2 inhibitors (SGLT2i) in addition to the SOC regimen (I, A).^[5]^ The Soluble guanylate cyclase stimulator (sGCs) is currently in clinical trials and has a different mechanism of action than other targeted cyclic guanosine monophosphate (cGMP) pathways.^[7,8]^ It can improve heart function and inhibit myocardial remodeling. The use of sGCs in clinical research has a lot of potentials.

The clinical efficacy of the 3 novel heart failure drugs is statistically significant, but there is a lack of direct comparison between the efficacy of the novel heart failure treatment regimen. This study will use network meta-analysis (NMA) to evaluate the different novel heart failure treatment regimens, to provide the evidence-based basis for clinical workers in the future.

## 2. Methods

### 2.1. Data sources and search strategy

Because the network meta-analysis is a secondary analysis study, it does not involve ethical approval. PubMed, The Cochrane Library, Embase, and Web of Science databases were randomized searched by computer to collect randomized controlled trials (RCTs) on the comparison of different anti-heart failure treatment regimens for patients with chronic heart failure from the establishment of the database to October 1, 2021. At the same time, reference literatures of published studies were traced back, and paper versions of relevant conferences were manually read to supplement. (Details of our search strategy are provided in the Supplementary Appendix, http://links.lww.com/MD2/B69)

### 2.2. Study selection

RCTs must be used in the studies. Patients with chronic heart failure who satisfied the clinical diagnostic criteria were over the age of 18. The follow-up period was at least 2 months. The experimental group received therapy with ARNI, SGLT2i, or sGCs. The SOC regimen, which included ACEI/ARB, beta-receptor antagonists, and MRA medication, was employed in the control group.

### 2.3. Data extraction and quality assessment

Two researchers independently screened studies, extracted data, and cross-checked them. Disputes, if any, shall be resolved through discussion or consultation with a third party. When screening studies, read the title first, then the abstract and entire text to determine whether to include it. To get information, contact the original study author by email or phone if necessary. The RCT bias risk assessment tool recommended by Cochrane Manual 5.1.0 was used to assess the risk of bias.

The following data were recorded: publication characteristics, countries or regions of the study, patient characteristics, New York heart association functional class, left ventricular ejection fraction, sample size, interventions, duration of follow-up, blinding, intention-to -treat analysis, and efficacy outcomes. The efficacy outcomes included rate of heart failure rehospitalization, all-cause mortality, cardiovascular mortality, rates of cardiovascular death or heart failure rehospitalization, the total symptom score on the kansas city cardiomyopathy questionnaire (KCCQ),^[9]^ and N-terminal pro-B-type natriuretic peptide (NT-proBNP).

### 2.4. Data analysis

A random-effect model was constructed based on frequency theory, and Stata16.0 software was used for direct and network meta-analysis. χ^2^ test and *I*^2^ value were used to determine heterogeneity. If there was significant heterogeneity between studies, the source of heterogeneity was analyzed first. The outcome indicators were odds ratio (OR) for dichotomous variables and mean difference (MD) for continuous variables, with a 95% confidence interval (CI) as the test level. If the number of included studies was greater than 10, a funnel plot was made to evaluate whether the intervention had a small sample effect and publication bias. The network plot represents the sample size and relationship of the interventions. When there was a closed loop, the node-splitting method was used to test the inconsistency. If the difference was not statistically significant, and the consistency model was used for analysis. At the same time, the node-splitting method was used to test the local inconsistency. By surface under the cumulative ranking (SUCRA), the advantages and disadvantages of therapies were quantitatively compared. The larger SUCRA was, the more likely the treatment was to become the best treatment. Then, the efficacy of different therapies could be compared comprehensively.

## 3. Results

### 3.1. Study selection

A total of 2830 related studies were obtained in the preliminary examination, and 17 RCTs were eventually included,^[10–26]^ including 38,088 patients (Study selection flow diagram inFig. 1).

**Figure 1.**
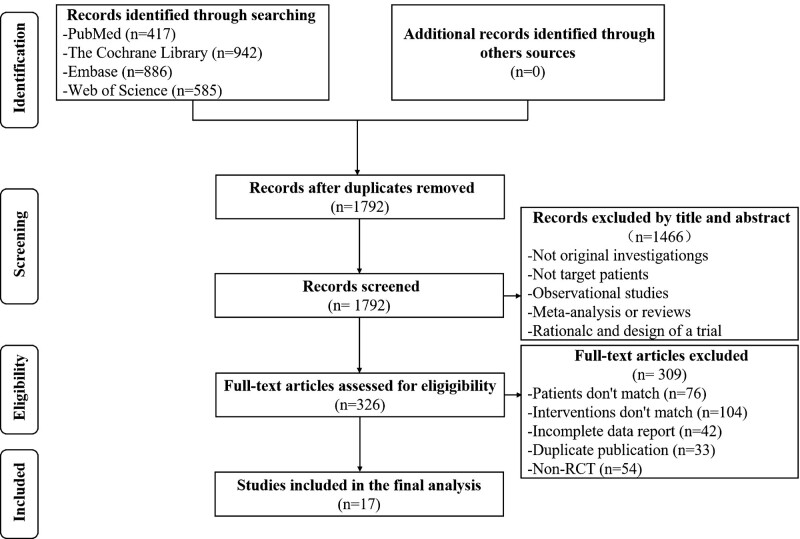
Study selection flow diagram.

### 3.2. Quality evaluation and baseline characteristics

**Figure 2.**
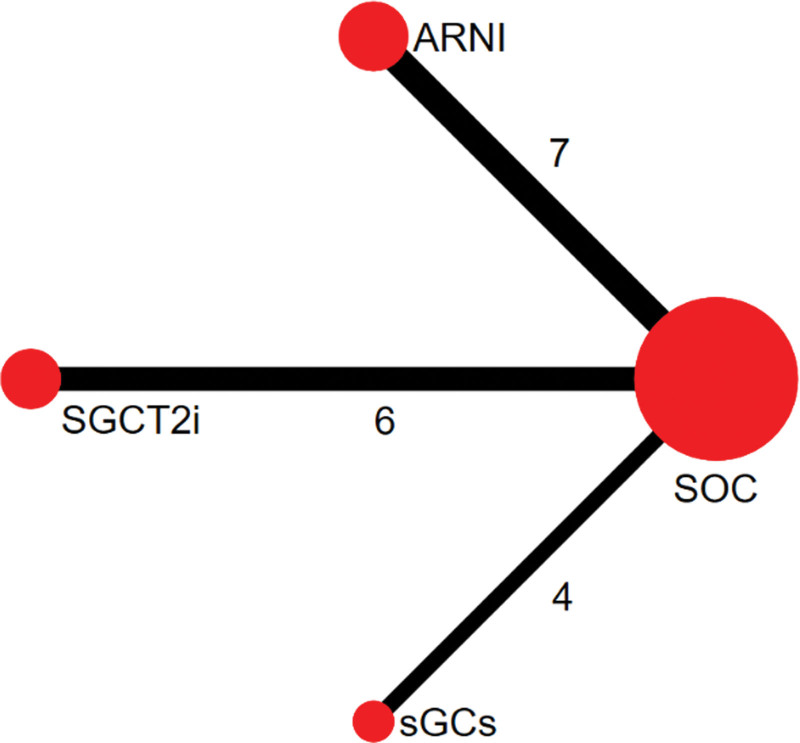
The network plot.

Random sequence generation, double-blind, allocation concealment, and complete outcome data were used in the majority of the studies (Quality evaluation inTable 1). A total of 4 interventions were included (Baseline characteristics in Table 2 and the network plot presented in Fig. 2). The SOC regimen included ACEI/ARB, β-receptor antagonists, and MRA drug therapy.^[10–26]^ The ARNI regimen included ARNI, β-receptor antagonists, and MRA drug therapy, and that there were 7 RCTs compared with the SOC regimen.^[10–16]^ The SGLT2i regimen included ACEI/ARB, β-receptor antagonists, MRA, and SGLT2i drug therapy, and that there were 6 RCTs compared with the SOC regime.^[17–22]^ The sGCs regimen included ACEI/ARB, β-receptor antagonists, MRA, and sGCs drug therapy, and that there were 4 RCTs compared with the SOC regime.^[23–26]^

**Table 1:** The result of the quality evaluation.

Study ID	Random method	Blinding	Allocation plan hidden	Integrity of the result data	Selective reporting	Other sources of bias
PARADIGM-HF 2014^[10]^	Computer	Double-blind	Not sure	Basically complete*, ITT	No	Not sure
PIONEER-HF 2019^[11]^	Computer	Double-blind	Interactive-response computer system	Basically complete*, ITT	No	Not sure
CURRENT 2019^[12]^	Patient order	Not sure	Not sure	Complete, ITT	No	Not sure
PRIME 2019^[13]^	Computer	Double-blind	Interactive-response computer system	Complete, ITT	No	Not sure
PARAMOUNT 2012^[14]^	Computer	Double-blind	Interactive-response computer system	Basically complete*, ITT	No	Not sure
PARAGON-HF 2019^[15]^	Not sure	Double-blind	Not sure	Basically complete*, ITT	No	Not sure
EVALUATE-HF 2019^[16]^	Not sure	Double-blind	Not sure	Basically complete*, ITT	No	Not sure
DEFINE-HF 2019^[17]^	Not sure	Double-blind	Not sure	Basically complete*, ITT	No	Not sure
DAPA-HF 2019^[18]^	Stratification	Double-blind	Interactive-response computer system	Basically complete*, ITT	No	Not sure
DECLARE-TIMI 58 2019^[19]^	Not sure	Double-blind	Not sure	Basically complete*, ITT	No	Subgroup analysis results
EMPIRE HF 2020^[20]^	Computer	Double blind	Pharmacy control	Basically complete*, ITT	No	Not sure
EMPEROR-REDUCED 2020^[21]^	Stratification	Double blind	Interactive-response computer system	Basically complete*, ITT	No	Not sure
EMPEROR-PRESERVED 2021^[22]^	Stratification	Double-blind	Not sure	Basically complete*, ITT	No	Not sure
SOCRATES-REDUCED 2015^[23]^	Not sure	Double-blind	Not sure	Basically complete*, ITT	No	Not sure
LEPHT 2013^[24]^	Not sure	Double-blind	Not sure	Basically complete*, ITT	No	Not sure
VICTORIA 2020^[25]^	Stratification	Double-blind	Not sure	Basically complete*, ITT	No	Not sure
SOCRATES-PRESERVED 2017^[26]^	Computer	Double-blind	Interactive-response computer system	Basically complete*, ITT	No	Not sure

ITT = intentional analysis.

* The study was lost to follow-up, but the number of lost to follow-up in each group was balanced, or the proportion of lost to follow-up was very low, which had little impact on the completeness of the result data.

**Table 2:** The result of the baseline characteristics.

						Interventions			
Study ID	Country/region	No patients (T/C)	Ejection fraction (%)	Average age (years)	Male ratio (%)	T	C	Follow-up time (months)	Outcome
PARADIGM-HF 2014 ^[10]^	47 countries	4187/4212	29.5 ± 6.2	63.8 ± 11.4	78.2	LCZ696	Enalapril	27	
PIONEER-HF 2019 ^[11]^	America	440/441	24.5 ± 5.5	62.0 ± 9.5	72.0	Sacubitril/Valsartan	Enalapril	2	
CURRENT 2019 ^[12]^	Taiwan	466/466	27.2 ± 7.0	61.8 ± 14.9	74.4	Sacubitril/Valsartan	Blank	15	
PRIME 2019 ^[13]^	Korea	60/58	33.8 ± 7.2	62.6 ± 11.0	61.1	Sacubitril/Valsartan	Valsartan	12	
PARAMOUNT 2012 ^[14]^	13 countries	149/152	58.0 ± 7.7	71.0 ± 9.2	43.5	LCZ696	Valsartan	9	
PARAGON-HF 2019 ^[15]^	43 countries	2407/2389	57.6 ± 8.0	72.8 ± 8.4	48.3	Sacubitril/Valsartan	Valsartan	9	
EVALUATE-HF 2019 ^[16]^	America	231/233	33.5 ± 10.0	67.3 ± 9.2	76.5	Sacubitril/Valsartan	Enalapril	3	
DEFINE-HF 2019 ^[17]^	America	131/132	26.5 ± 8.1	61.3 ± 11.5	73.3	Dapagliflozin	Placebo	3	
DAPA-HF 2019 ^[18]^	20 countries	2373/2371	31.1 ± 6.8	66.3 ± 10.9	76.6	Dapagliflozin	Placebo	18	
DECLARE-TIMI 58 2019 ^[19]^	33 countries	852/872	-	64.0 ± 6.8	62.6	Dapagliflozin	Placebo	50	
EMPIRE HF 2020 ^[20]^	Denmark	95/95	30.0 ± 5.0	63.5 ± 8.0	85.0	Empagliflozin	Placebo	3	
EMPEROR-REDUCED 2020 ^[21]^	20 countries	1863/1867	27.5 ± 6.0	66.8 ± 11.0	76.0	Empagliflozin	Placebo	16	
EMPEROR-PRESERVED 2021 ^[22]^	23 countries	2997/2991	54.3 ± 8.8	71.9 ± 9.6	55.4	Empagliflozin	Placebo	26	
SOCRATES-REDUCED 2015 ^[23]^	Europe, North America and Asia	91/92	29.0 ± 8.4	68 ± 12.5	82.0	Vericiguat	Placebo	4	
LEPHT 2013 ^[24]^	18 countries	67/69	27.8 ± 0.7	59.1 ± 12.0	85.0	Riociguat	Placebo	4	
VICTORIA 2020 ^[25]^	42 countries	2526/2524	28.9 ± 8.3	67.4 ± 12.2	76.0	Vericiguat	Placebo	11	
SOCRATES-PRESERVED 2017 ^[26]^	Europe, North America and Asia	96/93	56.5 ± 61.0	73.5 ± 9.5	52.4	Vericiguat	Placebo	4	

C = control group, T = test group; <inline-graphic xlink:href="fx1"/> heart failure rehospitalization rate; <inline-graphic xlink:href="fx2"/> all-cause mortality; <inline-graphic xlink:href="fx3"/> cardiovascular mortality; <inline-graphic xlink:href="fx4"/> rates of cardiovascular death or heart failure rehospitalization; <inline-graphic xlink:href="fx5"/> KCCQ score; <inline-graphic xlink:href="fx6"/> NT-proBNP.

### 3.3. Data consistency and inconsistency test

There was no closed loop between the 4 interventions, so the consistency test could only be performed. The results of the direct meta-analysis were close to those of network meta-analysis, indicating that the data was reliable (The result of direct and network meta-analysis in Table 3).

**Table 3:** The result of direct and network meta-analysis.

			Heterogeneity test			
Outcome	Interventions	Number of studies	P value	I2 value	Direct meta-analysis OR/MD (95% CI)	Network meta-analysis OR/MD (95% CI)
Heart failure rehospitalization rate	ARNI vs SOC	6^[10–15]^	.24	25%	0.75 (0.66, 0.84)*	0.77 (0.71, 0.83)*
	SGLT2i vs SOC	5^[17,18,20–22]^	.96	0%	0.70 (0.63, 0.77)*	0.70 (0.63, 0.77)*
	sGCs vs SOC	4^[23–26]^	.53	0%	0.88 (0.78, 0.99)*	0.88 (0.78, 0.99)*
	ARNI vs SGLT2i	0	–	–	–	1.10 (0.97, 1.25)
	ARNI vs sGCs	0	–	–	–	0.87 (0.75, 1.01)
	SGLT2i vs sGCs	0	–	–	–	0.79 (0.68, 0.93)
All-cause mortality	ARNI vs SOC	6^[10–15]^	.06	52%	0.80 (0.65, 0.98)*	0.81 (0.66, 0.99)*
	SGLT2i vs SOC	4^[17,18,21,22]^	.44	0%	0.92 (0.83, 1.01)	0.91 (0.77, 1.08)
	sGCs vs SOC	4^[23–26]^	.76	0%	0.93 (0.78, 1.10)	0.92 (0.69, 1.24)
	ARNI vs SGLT2i	0	–	–	–	0.89 (0.68, 1.16)
	ARNI vs sGCs	0	–	–	–	0.87 (0.61, 1.25)
	SGLT2i vs sGCs	0	–	–	–	0.98 (0.70, 1.38)
Cardiovascular mortality	ARNI vs SOC	4^[10,12,14,15]^	.06	60%	0.77 (0.61, 0.97)*	0.80 (0.70, 0.92)*
	SGLT2i vs SOC	4^[17,18,21,22]^	.84	0%	0.87 (0.78, 0.97)*	0.87 (0.76, 0.99)*
	sGCs vs SOC	4^[23–26]^	.76	0%	0.91 (0.75, 1.10)	0.91 (0.72, 1.14)
	ARNI vs SGLT2i	0	–	–	–	0.92 (0.76, 1.11)
	ARNI vs sGCs	0	–	–	–	0.88 (0.68, 1.15)
	SGLT2i vs sGCs	0	–	–	–	0.96 (0.74, 1.25)
Rates of cardiovascular death or heart failure rehospitalization	ARNI vs SOC	6^[10–15]^	.20	31%	0.74 (0.66, 0.83)*	0.76 (0.71, 0.82)*
	SGLT2i vs SOC	6^[17–22]^	.94	0%	0.76 (0.70, 0.82)*	0.76 (0.70, 0.82)*
	sGCs vs SOC	4^[23–26]^	.59	0%	0.87 (0.78, 0.97)*	0.87 (0.78, 0.97)*
	ARNI vs SGLT2i	0	–	–	–	1.01 (0.91, 1.13)
	ARNI vs sGCs	0	–	–	–	0.88 (0.77, 1.01)
	SGLT2i vs sGCs	0	–	–	–	0.87 (0.76, 1.00)*
KCCQ score	ARNI vs. SOC	2^[15,16]^	.13	51%	1.55 (0.34, 2.77)*	1.43 (0.43, 2.42)*
	SGLT2i vs. SOC	5^[17,18,20–22]^	.26	24%	1.89 (1.17, 2.61)*	1.88 (1.12, 2.65)*
	sGCs vs SOC	0	–	–	–	–
	ARNI vs SGLT2i	0	–	–	–	-0.46 (-1.69, 0.78)
	ARNI vs sGCs	0	–	–	–	–
	SGLT2i vs sGCs	0	–	–	–	–
NT-proBNP	ARNI vs SOC	3^[11,14,16]^	.02	74%	-65.75 (-191.80, 60.30)	-58.90 (-166.98, 49.17)
	SGLT2i vs SOC	4^[17,18,20,21]^	.23	30%	-140.39 (-222.18, -58.60)*	-134.63 (-237.70, -31.56)*
	sGCs vs SOC	0	–	–	–	–
	ARNI vs SGLT2i	0	–	–	–	75.73 (-75.91, 227.37)
	ARNI vs sGCs	0	–	–	–	–
	SGLT2i vs sGCs	0	–	–	–	–

* The difference was statistically significant.

ARNI = angiotensin receptor neprilysin inhibitors, SGLT2i = sodium-glucose cotransporter 2 inhibitors, KCCQ = Kansas city cardiomyopathy questionnaire, sGCs = soluble guanylate cyclase stimulators, SOC = standard-of-care (the traditional golden triangle).

### 3.4. Network meta-analysis

#### 3.4.1. Heart failure rehospitalization rate.

**Figure 3.**
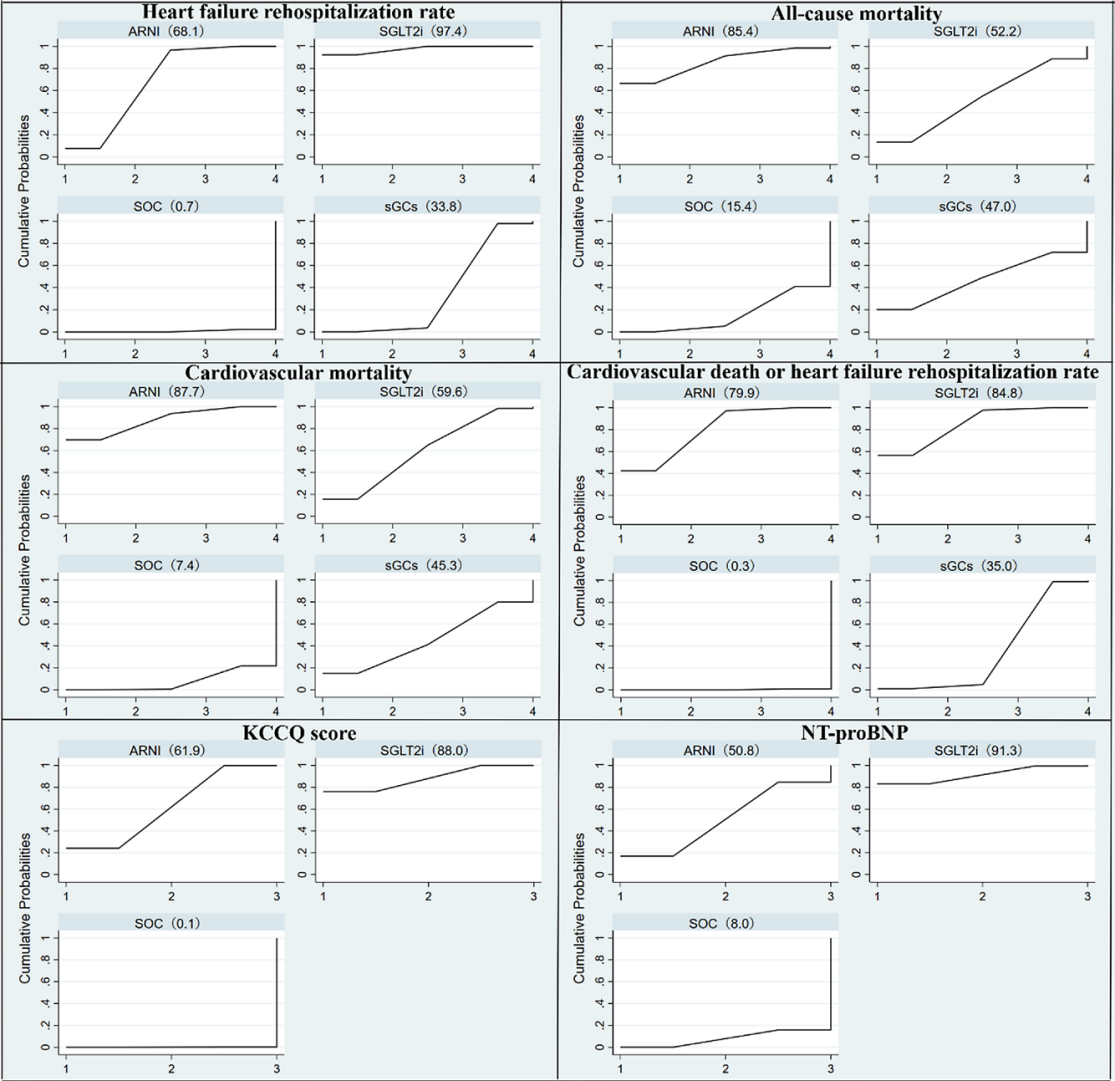
The result of SUCRA sequencing.

A total of 15 RCTs were included.^[10–15,17,18,20–26]^ Results of network meta-analysis showed that ARNI was lower than SOC (OR = 0.77, 95% CI: 0.71–0.83). SGLT2i was lower than SOC (OR = 0.70, 95%CI: 0.63-0.77). sGCs was lower than SOC (OR = 0.88, 95% CI: 0.78–0.99). SGLT2i was also lower than sGCs (OR = 0.79, 95%CI: 0.68–0.93). There was no statistical significance among other interventions (Table 3). SUCRA sequencing results showed that: SGLT2i (97.4) > ARNI (68.1) > sGCs (33.8) > SOC (0.7) (Fig. 3).

#### 3.4.2. All-cause mortality.

A total of 14 RCTs were included.^[10–15,17,18,21–26]^ Results of network meta-analysis showed that ARNI was lower than SOC (OR = 0.81, 95% CI: 0.66–0.99). There was no statistical significance among other interventions (Table 3). SUCRA sequencing results showed that: ARNI (85.4) > SGLT2i (52.2) > sGCs (47.0) > SOC (15.4) (Fig. 3).

#### 3.4.3. Cardiovascular mortality.

A total of 12 RCTs were included.^[10,12,14,15,17,18,21–26]^ The ARNI and SOC comparison included 4 RCTs with slightly higher heterogeneity. Due to the limited number of included RCTs, analyses could only be excluded one by one. Heterogeneity was found to be slightly lower when the CURRENT study ^[12]^ was excluded (*P* = .24, *I*^2^ = 30%). It is speculated that the CURRENT study is likely to use a blank control. Of course, there are other possibilities. Due to the limitation of the small number of included RCTs, random-effects model was used for analysis. Results of network meta-analysis showed that ARNI was lower than SOC (OR = 0.80, 95% CI: 0.70–0.92), and SGLT2i was also lower than SOC (OR = 0.87, 95% CI: 0.76–0.99). There was no statistical significance among other interventions (Table 3). SUCRA sequencing results showed that: ARNI (87.7) > SGLT2i (59.6) > sGCs (45.3) > SOC (7.4) (Fig. 3).

#### 3.4.4. Rates of cardiovascular death or heart failure rehospitalization.

A total of 16 RCTs were included.^[10–15,17–26]^ Results of network meta-analysis showed that ARNI was lower than SOC (OR = 0.76, 95% CI: 0.71–0.82). SGLT2i was lower than SOC (OR = 0.76, 95% CI: 0.70–0.82). sGCs was lower than SOC (OR = 0.87, 95% CI: 0.78–0.97). SGLT2i was also lower than sGCs (OR = 0.87, 95% CI: 0.76–1.00). There was no statistical significance among other interventions (Table 3). SUCRA sequencing results showed that: SGLT2i (84.8) > ARNI (79.9) > sGCs (35.0) > SOC (0.3) (Fig. 3).

#### 3.4.5. The total symptom score on the KCCQ.

A total of 7 RCTs were included.^[15–18,20–22]^ Results of network meta-analysis showed that ARNI was superior to SOC (MD = 1.43, 95% CI: 0.43–2.42), and SGLT2i was also superior to SOC (MD = 1.88, 95% CI: 1.12–2.65). There was no statistical significance among other interventions (Table 3). SUCRA sequencing results showed that: SGLT2i (88.0) > ARNI (61.9) > SOC (0.1) (Fig. 3).

#### 3.4.6. NT-proBNP.

A total of 7 RCTs were included.^[11,14,16–18,20,21]^ Results of network meta-analysis showed that SGLT2i was superior to SOC (MD = −134.63, 95% CI: −237.70 to −31.56). There was no statistical significance among other interventions (Table 3). SUCRA sequencing results showed that: SGLT2i (91.3) > ARNI (50.8) > SOC (8.0) (Fig. 3).

#### 3.4.7. Risk assessment of bias.

The funnel plot was drawn for publication bias test for the outcome index of cardiovascular death or heart failure rehospitalization. The results showed that the distribution of each study point was roughly symmetrical on both sides of the funnel plot, suggesting that there was little possibility of publication bias (The funnel plot inFig. 4).

**Figure 4.**
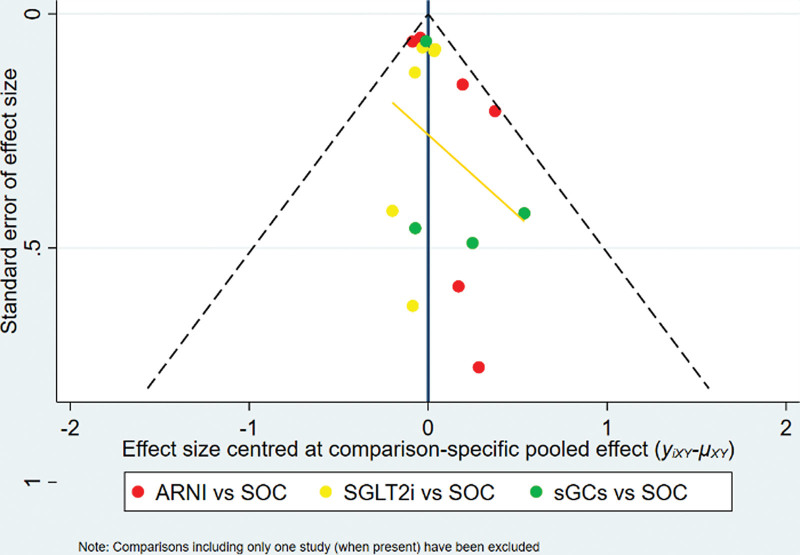
The funnel plot.

## 4. Discussion

The representative drug of ARNI is sacubitril and valsartan sodium, which has good safety and tolerability. Compared with the use of ACEI drugs, this drug does not increase the incidence of adverse reactions such as serious angioedema, renal impairment, hyperkalemia, and coughing. The effect on basal heart rate and blood creatinine is small, and the effect of blood pressure control is better.^[27]^ The results of this network meta-analysis showed that among the 4 interventions, the ARNI regimen was the most effective in improving the overall prognosis of heart failure, especially in reducing all-cause mortality or cardiovascular mortality. Although the ARNI regimen could significantly improve heart function, there was no statistically significant difference from the SOC regimen in reducing the outcome indicators of NT-proBNP. It may be related to the increased levels of natriuretic peptide, bradykinin, adrenomedullin, and other endogenous vasoactive peptides in its enkephalinase inhibitors.^[28]^

SGLT2i is not only a new type of hypoglycemic agent, but also has a high value in the treatment of cardiovascular diseases. In addition to increasing urine glucose, reducing blood sugar, diuresis, and related hemodynamic effects, SGLT2i also has effects on myocardial metabolism, iontransporters, fibrosis, adipokines, and vascular function, thereby improving the prognosis of heart failure.^[29,30]^ The results of the network meta-analysis showed that among the 4 interventions, the SGLT2i regimen had the best effect on the KCCQ score and NT-proBNP, and might be better than the ARNI regimen in reducing the rate of heart failure rehospitalization. The SGLT2i regimen had the most significant effect in improving the symptoms of heart failure and improving the quality of life. It may be inseparable from the effect of SGLT2i drugs on blood volume. Urinary sugar excretion reduces blood sugar, while reducing volume and blood pressure through osmotic diuresis and increasing diuretic sensitivity, without the adverse effects of increased heart rate caused by decreased blood volume. SGLT2i reduces the load on the heart and kidneys and improves the quality of life.^[31]^

Vericiguat is a new type of oral soluble guanylate cyclase stimulator with a dual mechanism of increasing cGMP. On the one hand, sGC can be directly stimulated by binding sites unrelated to NO, and on the other hand, sGC can be sensitive to endogenous NO by stabilizing the NO-sGC binding sites, thus having a multi-dimensional protective effect on the heart.^[32]^ The results of this network meta-analysis showed that among the 4 interventions, the efficacy of the sGCs regimen was slightly inferior to ARNI and SGLT2i regimens. In terms of reducing cardiovascular death or heart failure rehospitalization rate and heart failure rehospitalization rate, the sGCs regimen was more effective than the SOC regimen, but it did not reduce the risk of death. The sGCs regimen has shown the effectiveness of the treatment of heart failure, but the mechanism of action and clinical trials of sGCs are still under study. More large-scale clinical trials are needed to verify in the future.

Some limitations of this study exist. First, in the included studies, the ejection fraction classification and heart failure comorbidities were different, which might affect the results of the study. Second, no closed loop was formed between the 4 interventions, which might affect the stability of the results. Third, in the outcome indexes of KCCQ Score and NT-proBNP, the number of studies that could be included was small, and the heterogeneity between them was slightly larger. Lastly, the length of follow-up of the included studies was different, which might have a certain impact on the long-term prognosis of heart failure.

Due to the influence of indirection of evidence and sample size, it is hoped that in the future there will be a direct comparison of these 3 novel drugs in randomized controlled trials to verify the relationship between their efficacy.

## 5. Conclusions

The available evidence suggests that all 3 novel heart failure drugs can improve the prognosis of heart failure, and the efficacy of the sGCs regimen may be inferior to the ARNI and SGLT2i regimens. The ARNI regimen may have the best efficacy in improving all-cause mortality and cardiovascular mortality. The ARNI and SGLT2i regimens have similar efficacy in improving cardiovascular death or heart failure rehospitalization rate and heart failure rehospitalization rate. The SGLT2i regimen may have the best curative effect in improving the KCCQ score and NT-proBNP outcome index.

## Author contributions

Conceptualization: Kai Tang

Data curation: Jianli Wu, Lin Luo

Formal analysis: Dejin Li

Investigation: Jiuju Ran

Methodology: Li Zhang

Project administration: Dan Wang

Resources: Dan Zhao

Software: Lin Luo, Min Yu, Xu Yang

Supervision: Lin Luo, Xu Yang

Validation: Anfang Chen, Lin Luo, Xu Yang

Visualization: Lin Luo

Writing – original draft: Lin Luo

Writing – review & editing: Lin Luo

## Supplementary Material


