# Supplementary material for: Efficacy of three novel drugs in the treatment of heart failure: A network meta-analysis
Source: Medicine (Baltimore). 2022 Jul 22;101(29):e29415. doi: 10.1097/MD.0000000000029415 (PMC9302283; doi:10.1097/MD.0000000000029415)
Supplement: Supplementary file 1 [file medi-101-e29415-s001.docx]

**Search strategy by PubMed**

#1　Heart Failure [Mesh Terms]

#2　Heart Failure [Title/Abstract] OR Cardiac Failure [Title/Abstract] OR HF [Title/Abstract]

#3　#1 OR #2

#4　Angiotensin Receptor Neprilysin Inhibitors [Title/Abstract] OR ARNI [Title/Abstract] OR Sacubitril/Valsartan [Title/Abstract] OR LCZ696 [Title/Abstract] OR Sodium-Glucose Cotransporter 2 Inhibitors [Title/Abstract] OR SGLT-2 Inhibitors [Title/Abstract] OR SGLT2i [Title/Abstract] OR Dapagliflozin [Title/Abstract] OR Empagliflozin [Title/Abstract] OR Soluble Guanylate Cyclase Stimulators [Title/Abstract] OR sGC Stimulators [Title/Abstract] OR sGCs [Title/Abstract] OR Vericiguat [Title/Abstract] OR Riociguat [Title/Abstract]

#5　Randomized Controlled Trial [Mesh Terms]

#6　Randomized Controlled Trial [Publication Type] OR Randomized Controlled Trial [All Fields] OR RCT [All Fields]

#7　#5 OR #6

#8　#3 AND #4 AND #7
